# Correlates of out-of-pocket and catastrophic health expenditures in Tanzania: results from a national household survey

**DOI:** 10.1186/1472-698X-14-5

**Published:** 2014-03-05

**Authors:** Ethel Mary Brinda, Rodriguez Antonio Andrés, Ulrika Enemark

**Affiliations:** 1 Department of Public Health, Section for Health Services Research and Health Promotion, Aarhus University, Aarhus C 8000, Denmark

**Keywords:** Tanzania, Catastrophic expenditures, Out-of-pocket payments, Traditional healers

## Abstract

**Background:**

Inequality in health services access and utilization are influenced by out-of-pocket health expenditures in many low and middle-income countries (LMICs). Various antecedents such as social factors, poor health and economic factors are proposed to direct the choice of health care service use and incurring out-of-pocket payments. We investigated the association of these factors with out-of-pocket health expenditures among the adult and older population in the United Republic of Tanzania. We also investigated the prevalence and associated determinants contributing to household catastrophic health expenditures.

**Methods:**

We accessed the data of a multistage stratified random sample of 7279 adult participants, aged between 18 and 59 years, as well as 1018 participants aged above 60 years, from the first round of the Tanzania National Panel survey. We employed multiple generalized linear and logistic regression models to evaluate the correlates of out-of-pocket as well as catastrophic health expenditures, accounting for the complex sample design effects.

**Results:**

Increasing age, female gender, obesity and functional disability increased the adults’ out-of-pocket health expenditures significantly, while functional disability and visits to traditional healers increased the out-of-pocket health expenditures in older participants. Adult participants, who lacked formal education or worked as manual laborers earned significantly less (p < 0.001) and spent less on health (p < 0.001), despite having higher levels of disability. Large household size, household head’s occupation as a manual laborer, household member with chronic illness, domestic violence against women and traditional healer’s visits were significantly associated with high catastrophic health expenditures.

**Conclusion:**

We observed that the prevalence of inequalities in socioeconomic factors played a significant role in determining the nature of both out-of-pocket and catastrophic health expenditures. We propose that investment in social welfare programs and strengthening the social security mechanisms could reduce the financial burden in United Republic of Tanzania.

## Background

Low and middle income countries (LMICs) support 84% of the global population, comprise 90% of the global disease burden and yet, account for only 12% of global resources spent on health [1]. Public expenditures on health, as a share of their Gross Domestic Product (GDP) are significantly less in LMICs [2,3]. Most of the health expenditures are paid directly as out-of-pocket (OOP) payments [4]. LMICs also experience *overlapping health transition*[5] with the additional burden of both infectious and chronic diseases.

The vast majority of African countries rely on direct OOP health expenditures and experiences a high burden of catastrophic health expenditures [6-8]. Tanzania with its growing ageing population, supports a current older population (60 years and above) of 1.5 million, which is projected to increase to 3 million by 2025 [9]. Tanzania spends a low share of GDP (7.2% ) on health, with a meagre public expenditure of 39% of the total health costs [10]. The OOP health expenditures account for 52% of total health spending, while various public and private pre-payment schemes contribute the remaining expenditures [11]. User fees at public health facilities were introduced in Tanzania in the 1990s with the intention of mobilizing resources and providing sustainable healthcare [12]. However, ineffective exemption systems, ancillary health care costs of the health seekers through transportation, food and accommodation escalates the high OOP health expenditures [13]. Health care seeking through private healthcare and faith-based health facilities or traditional healers contributes to most of the health service utilization [14]. These providers charge user fees, have some waiver system and do not provide exemptions [12].

Affordability [15] and accessibility [4] determine health service utilization in many LMICs. The use of essential health services are reported to be avoided by poor people with the increasing demand for OOP health expenditures [16,17]. Andersons’ behavioral model aids the theoretical understanding of the nature of health service utilization and subsequent OOP health expenditures in LMICs [3,18]. High OOP health expenditures have a serious impact on vulnerable people who subsequently experience debt, income loss and catastrophic health expenditures [7,19]. Health expenditures are regarded catastrophic, when they exceed 40% of household’s effective income remaining after subsistence needs [20].

Knowledge about the determinants of OOP expenditure on health is vital from a health policy perspective, to inform the design of interventions or system changes that ensure accessible and financially protective health services to vulnerable people. The studies on determinants of OOP health expenditure in various LMICs are primarily focused on adult populations. The research findings cannot necessarily be extrapolated to older people who differ in disease patterns [21], health-seeking behaviors [22] and access to resources in LMICs [10]. Moreover, the relative absence of social welfare policies and inequitable access to health services require the specific assessment of OOP health expenditures prevalent in LMICs [4,23]. Hence, we aim to investigate the determinants influencing OOP health expenditures among the adult as well as the older population aged above 60 years in Tanzania. We also intend to explore the determinants of catastrophic health expenditures based on Tanzanian households’ non-food expenditures.

## Methods

### The Tanzania National Panel survey

We employed a cross-sectional study design. This study uses secondary data from the first round of Tanzania National Panel Survey (TZNPS), implemented by National Bureau of Statistics of United Republic of Tanzania [24]. The first wave of the survey was conducted between October 2008 and October 2009. TZNPS employed multistage, stratified, random sampling to generate nationally representative samples. The first stage of sampling units comprised 410 clusters of enumeration areas in urban and rural areas. The second stage involved 3265 households. All members of the selected households were invited and those who provided verbal informed consent, were included in the study. The final sample included 8297 participants, of which 7279 were aged between 18 and 59 years and 1018 participants were above 60 years. The details of the participants’ sampling information are available elsewhere [24]. To obtain individual and household level data, trained field staffs conducted person-to-person interviews employing structured questionnaires and recorded anthropometric measures [24]. At the individual level, the data included socioeconomic characteristics, self-reported disabilities, health service visits and OOP health expenditures by type of health facilities. Data on household characteristics, availability of durable assets, household’s total health expenditures and basic facilities like access to safe drinking water and toilet facilities for the past 4 weeks were recorded. The OOP health expenditures included the utilization of private outpatient clinics, preventive health services, over-the counter medications and use of traditional healer services.

### Variable specification

Based on Anderson’s model, the factors determining the health care demand were categorized into predisposing, enabling and need factors. We included age, gender, marital and occupation status variables as predisposing factors; education, asset index as proxy measures for wealth were considered as enabling factors to OOP health expenditures. The asset index was estimated using eighteen variables, which provided information on housing characteristics, type of water supply, sanitation in the household, and ownership of the household’s durable assets. Obesity, presence of visual defects, hearing defects, limb defects, self-reported functional disabilities and psychological morbidity were included as need variables.

### Statistical analyses

We initially analyzed the study variables using descriptive statistics. We used the statistical technique, Principal Component Analysis (PCA) to construct the asset index [25]. We performed Kaiser-Meyer-Olkin (KMO) measure of sampling adequacy. The obtained value of 0.80 was liable for conducting PCA. Scoring factors were derived from the first principal component, which was a linear index of variables with common information, and this score was used to construct the household’s asset index. We used Mann–Whitney U and Chi-square test to assess the bivariate associations between the study variables. Our outcome variable OOP health expenditure was typically non-parametric and positively skewed with influential outliers. Traditional ordinary least square regressions with log-transformation and retransformations are too inconsistent to handle skewness and provide inferences in natural units of mean expenditures [26]. Generalized linear regression models (GLM) are flexible to model skewed expenditure data and avoid the issue of outcome transformation [27]. Survey GLM with gamma distribution and log link function [28,29], was employed to assess various determinants of OOP health expenditures and account for the complex survey design.

To analyse the determinants of catastrophic health expenditure, we used non-food expenditures of the household as a close measure for the household’s capacity to pay. We estimated the share of OOP health expenditure in the household’s non-food expenditure (E_*j*_) to define catastrophic health expenditure:

$$E_{j}=HE_{j}/\mathrm{NF}E_{j}*100\;$$

Where, HE_*j*_ is the household’s average monthly OOP health expenditure; NFE_*j*_ denotes the household’s average monthly non-food expenditure. We dichotomized and coded the outcome as ‘1’ , when E_*j*_ exceeded the threshold of 40% [7], ‘0’ if otherwise. We employed survey logit model to assess the determinants of catastrophic health expenditure in households. We performed all analyzes using statistical software STATA 12.1.

## Results

We present the socio-demographic and health profiles of the study participants in Table 1. In the following, the study participants aged between 18–59 years are referred to as adult participants and those aged 60 years and above are termed older participants.

**Table 1:** Socio-demographic and health profiles of the participants

**Variables**	**Adult population (n = 7279)**	**Older population (n = 1018)**
	**Percentage/Mean (SD)**	**Percentage/Mean (SD)**
Age in years	37.7 (16.5)	70.5 (8.1)
Gender:		
Male	46.5	46.9
Female	53.5	53.0
Marital status:		
Single/Married	86.0	58.9
Widowed/separated	14.0	41.1
Education:		
No formal education	24.3	60.3
Primary education	61.3	34.3
Secondary education	12.8	4.2
Higher secondary education	0.7	0.1
Graduate	0.9	1.0
Occupation status:		
Manual laborers	53.5	67.3
Others	46.5	32.7
Lack of access to basic facilities:		
No access to safe drinking water	77.2	80.6
No access to toilet facilities	9.6	6.9
Presence of disabilities:		
Physical disabilities^a^	4.3	12.6
Psychiatric disorders	0.4	0.4
Functional disabilities	4.9	25.7
Body Mass Index^b^:		
Men	22.0 (5.4)	21.7 (4.2)
Women	23.2 (4.9)	22.3 (5.2)
OOP health expenditures (in US dollars)	2.2 (9.5)	0.7 (6.3)
Self-reported monthly income (in US dollars)	92.3 (260.3)	19.1 (178.2)

^a^Physical disablity includes presence of visual impairment, hearing or limb defects; ^b^Body Mass Index in Kg/m^2^.

### Participant characteristics

A total of 7279 adults and 1018 older participants were included in the present study. Majority of the study participants were women. Most of the adult participants had primary education (86%), while only 14% had more than primary education. Among the older participants, the majority were women, lacked formal education and had past occupation as manual laborers. Only 35% of households had safe drinking water access, while 92% households had access to toilet facilities. The average family size of the households was 5.1 (SD 2.8).

### Nature of resources and needs among study participants

The self-reported monthly income differed significantly between the adults and older participants (Mann- Whitney U = 3442425; p < 0.001). Adult women (U = 5559127.0; p < 0.001) and those who worked as manual laborers (U = 5328429.5; p < 0.001) had a significantly lower income. Older women had a significant lower income compared to older men (U = 115054.0; p < 0.001). Adult women (*χ*^2^ = 6.3; p < 0.001) and adult participants (*χ*^2^ = 5.4; p < 0.001) who lacked formal education reported higher disability. However, older women did not report higher disabilities (*χ*^2^ = 2.8; p = 0.09).

### Nature of health service utilization and OOP health expenditure

About 1030 (14.2%) of the adult participants had visited medical health services and 117 participants (1.6%) had visited traditional healers within the past month. Older participants, who visited medical health services, were 190 (18.7%). The adult participants utilized traditional healers or faith-based services significantly more than the older participants (*χ*^2^ = 5.7; p = 0.01). However, private healthcare utilization did not significantly differ among the adults and older participants (*χ*^2^ = 3.6; p = 0.06). The mean OOP health expenditures among the adult participants, over the past month was 2.2 (SD 9.5) US$, while the older participants’ OOP health expenditure was 0.7 (SD 6.3) US$. Total OOP expenditures were spent mostly on health professional fees 0.8 (SD 7.5) US$, followed by over -the-counter medications 0.2 (SD 1.1) US$ and hospital admissions 0.1 (SD 3.6) US$.

### Correlates of OOP health expenditure among adult participants

We present the bivariate and multivariate analyses for the correlates of adult OOP health expenditures in Table 2. Factors such as female gender, occupation as unskilled manual laborer, hearing defects and functional disability were significantly associated with higher adult OOP health expenditures. Traditional healer visits increased the OOP health expenditures among the adult participants. The higher OOP health expenditure among women could be attributed to expenditure on their reproductive health, because women aged above 45 years, had significantly less OOP health expenditures (β = −0.37; 95% CI −0.68, −0.04; p = 0.03), than men.

**Table 2:** Factors associated with out-of-pocket health expenditure among adult participants (18yrs-59yrs) (n = 7279)

**Explanatory variables**	**Bivariate statistics**^**a**^		**Multivariate statistics**^**b**^	
	**β (95% CI)**	**p value**	**β (95% CI)**	**p value**
Age in years	+0.02 (+0.01; +0.04)	<0.001	+0.03 (+0.02; +0.05)	<0.001
Female gender	+0.42 (+0.15; +0.68)	0.002	+0.60 (+0.34; +0.86)	<0.001
Lack of formal education	−0.46 (−0.80; −0.12)	0.007	−0.33 (−0.72; +0.06	0.10
Widowhood	+0.14 (−0.29; +0.58)	0.51	−0.29 (−0.69; +0.09)	0.13
Working as manual laborer	−0.64 (−0.91; −0.37)	<0.001	−0.62 (−0.86; −0.39)	<0.001
Asset Index^c^	+0.03 (−0.02; +0.07)	0.26	+0.02 (−0.02; +0.06)	0.27
BMI more than 30 (kg/m^2^)	+0.99 (+0.42; +1.55)	<0.001	+0.55 (−0.08; +1.18)	0.09
Presence of blindness/visual defect	+0.01 (−0.51; +0.53)	0.96	−0.12 (−0.74; +0.51)	0.71
Presence of hearing defect	+1.25 (−0.20; +2.69)	0.09	+1.96 (+0.24; +3.68)	0.02
Presence of limb defect	+0.93 (+0.36; +1.50)	<0.001	+0.51 (−0.37; +1.39)	0.25
Presence of psychiatric morbidity	−0.62 (−1.72; +0.48)	0.27	+0.41 (−1.18, +1.98)	0.61
Presence of functional disability	+1.77 (+1.28; +2.25)	<0.001	+1.08 (+0.56; +1.61)	<0.001
Traditional healer visits	+1.66 (+1.16; +2.16)	<0.001	+1.47 (+1.23; +1.81)	<0.001

^a^Survey generalized linear regression models with log link function using out-of-pocket health expenditure (in Tanzanian Schilling) as the dependent variable for adult participants aged between 18 and 59 years; ^b^Adjusted for all bivariate significant variables with out-of-pocket health expenditure (in Tanzanian Schilling) as the dependent variable; ^c^Asset Index constructed using Principal Component Analysis. BMI = Body Mass Index.

### Correlates of OOP health expenditure among older participants

We present the bivariate and multivariate analyses for the factors associated with OOP health expenditures among the older participants in Table 3. Presence of visual impairment, Functional disability and traditional healthcare visits significantly increased the OOP health expenditure, after adjusting for potential confounders. Older participants with occupations as unskilled manual laborer spent significantly less out-of their pockets, despite a higher prevalence of disability. Among the older participants aged above 65 years (n = 744), unskilled manual laborers (β = −0.71; 95% CI −1.30, −0.12; p = 0.01) and those who lacked formal education (β = −0.62; 95% CI −1.21, −0.02; p = 0.04) had lower OOP health expenditures. While, factors such as functional disability (β = +0.89; 95% CI +0.32, +1.46; p = 0.02), and visits to traditional healers (β = +1.40; 95% CI +0.41, +2.39; p = 0.006), increased their OOP health expenditures.

**Table 3:** Factors associated with out-of-pocket health expenditure among older participants (>60yrs) (n = 1018)

**Explanatory variables**	**Bivariate statistics**^**a**^		**Multivariate statistics**^**b**^	
	**β (95% CI)**	**p value**	**β (95% CI)**	**p value**
Age in years	+0.01 (−0.02; +0.04)	0.64	−0.01 (−0.04; +0.02)	0.42
Female gender	−0.35 (−0.90; +0.19)	0.20	−0.37 (−0.89, +0.16)	0.16
Lack of formal education	−0.69 (−1.23; −0.16)	0.01	−0.48 (−0.96, +0.01)	0.05
Widowhood	−0.13 (−0.68; +0.42)	0.63	−0.26 (−0.73; +0.22)	0.28
Worked as manual laborer in the past	−0.73 (−1.31; −0.15)	0.01	−0.29 (−0.90; +0.30)	0.33
Asset Index^c^	+0.03 (−0.04; +0.09)	0.39	+0.06 (−0.01; +0.12)	0.08
BMI more than 30 (kg/m^2^)	+0.87 (+0.26; +1.49)	0.005	+0.62 (−0.04; +1.28)	0.06
Presence of blindness/visual defect	+0.85 (+0.22; +1.49)	0.009	+1.03 (+0.29; +1.77)	0.01
Presence of hearing defect	−0.61 (−1.64; +0.43)	0.25	−0.24 (−1.35; +0.86)	0.66
Presence of limb defect	+0.11 (−0.81; +1.04)	0.81	−0.14 (−1.04; +0.75)	0.75
Presence of psychiatric morbidity	−1.16 (−2.60; +0.27)	0.11	−0.98 (−2.56; +0.60)	0.22
Presence of functional disability	+1.05 (+0.53; +1.57)	<0.001	+0.70 (+0.14; +1.26)	0.01
Traditional healer visits	+1.67 (+0.69; +2.65)	<0.001	+1.43 (+0.66; +2.20)	<0.001

^a^Survey generalized linear regression models with log link function using out-of-pocket health expenditure (in Tanzanian Schilling) as the dependent variable for older participants aged above 60years; ^b^Adjusted for all bivariate significant variables with out-of-pocket health expenditure (in Tanzanian Schilling) as the dependent variable ; ^c^Asset Index constructed using Principal Component Analysis. BMI = Body Mass Index.

### Correlates of Catastrophic health expenditure in households

The proportion of households that experienced catastrophic health expenditure was 18%. We present the bivariate and multivariate analyses for the correlates of the households’ catastrophic health expenditure in Table 4. During the multivariate analysis, factors such as household members with chronic illnesses, household head’s occupation as a manual laborer, healthcare visits to a traditional healer, a household size of more than five and domestic violence against women significantly increased the likelihood of experiencing catastrophic health expenditure, after adjusting for all bivariate significant variables. Wealthy households were spared catastrophic health expenditure.

**Table 4:** Factors associated with catastrophic health expenditure in the households of Tanzania (n = 3265)

**Explanatory variables**	**Bivariate statistics**^**a**^		**Multivariate statistics**^**b**^	
	**OR (95% CI)**	**p value**	**OR (95% CI)**	**p value**
Age(in years) of the household head	1.00 (0.99, 1.01)	0.06	1.00 (0.99, 1.01)	0.14
Women being household head	1.04 (0.84, 1.27)	0.73	1.05 (0.85, 1.30)	0.61
Household head without formal education	1.26 (0.97, 1.64)	0.08	0.80 (0.59, 1.08)	0.15
Household head being manual laborer	1.75 (1.28, 2.39)	<0.001	1.54 (1.11, 2.12)	0.01
Household size more than 5	2.15 (1.77, 2.61)	<0.001	1.68 (1.37, 2.07)	<0.001
Asset Index^c^	0.83 (0.67, 1.03)	0.09	0.78 (0.62, 0.98)	0.03
Household member with functional disability	1.61 (1.29, 2.02)	0.04	1.19 (0.93, 1.51)	0.15
Violence against women	1.80 (1.36, 2.39)	<0.001	1.41 (1.05, 1.91)	0.02
Household member affected with chronic disease	2.01 (1.50, 2.68)	<0.001	1.92 (1.43, 2.58)	<0.001
Traditional healer visits of the household	5.17 (3.01, 8.86)	<0.001	3.38 (1.96, 5.81)	<0.001

^a^Survey logistic regression models with catastrophic health expenditure as the dependent variable; ^b^Adjusted for bivariate significant covariates with, catastrophic health expenditure as the dependent variable; ^c^Asset Index constructed with Principal Component Analysis, coded as 0 = low household asset index, 1 = high household asset index.

## Discussion

Our study evaluated the nature and correlates of OOP health expenditures among adult and older participants in the United Republic of Tanzania. Its strengths include a relatively larger sample size, representative sampling and studying a combined model of social as well as health correlates. However, self-reported healthcare variables risk the possibility of recall and response bias. Cross-sectional nature of this study prevents establishing any causal associations. Unavailability of data on disease variables to explain the need for OOP health expenditures can be a potential limitation of this study.

### Correlates of OOP health expenditure among adults and older people

Women of reproductive age had a significantly higher OOP expenditure than the older women in our study. This is in line with a Tanzanian study, which reported the prevalence of high informal payments and increased need for health service utilization among younger women and their children [30]. With the meagre resources and a higher prevalence of disability, these women are at great risk of being confronted with the financial burden of OOP health expenditures. Many people rely on the services of traditional healers and face increased out-of-pocket health expenditure in Africa [22,31]. Our findings show that OOP costs for traditional healers are high among the adult participants. Culturally ingrained beliefs for various illnesses and inconsistencies in health service access urge them to seek the traditional healers with high OOP expenses [32]. Akin to earlier studies, obesity [33] and disability [34,35] were also associated with higher OOP health expenditures in our study population. Disabilities during youth may be attributable to poor health status, related poor nutrition, living conditions and a high incidence of infectious diseases. Disabilities due to physical, functional, and psychiatric morbidities can affect the nature of health service utilization and demand high OOP health expenses among older people. A higher prevalence of disabilities among elderly are known to be associated with increased OOP health expenditure in LMICs [36]. Economic disadvantages related to the older peoples’ disabilities can exacerbate their untreated medical illness, increase dependency and restrict their access to health services. Due to the absence of social security systems, the older people either suffer financial burden through OOP payments or remain disabled without seeking health care.

### Economic inequality and OOP health expenditure

The socioeconomic status of both adult and older participants was inversely associated with OOP health expenditures in our study. African studies [37,38] have consistently reported the differences in health care utilization and expenditure in terms of socioeconomic status. A study from Tanzania [39] also found that, occurrence of higher health care seeking and subsequent expenditure among the people with a higher socioeconomic status. OOP health expenditures remain an obstacle for health service utilization, and consistently increase the burden of diseases among people with poor literacy and low income laborers compared to the more affluent. Economic deprivation worsens the health status of these individuals and leads to decreased labor productivity [19]. The vicious cycle of further economic loss and increased vulnerability to illnesses predisposes to catastrophic health expenditures [40].

### Resources and need for OOP health expenditure

OOP expenditure depends on the resources and healthcare needs of an individual. These resources and needs have a complex bidirectional interaction. The rich have better living conditions and better health, thereby reducing their needs for OOP health expenditure. However, they are endowed with better economic resources, education, enhanced awareness towards the need for health care and the thresholds at which they access that service are correspondingly lower. On the contrary, the poor and those with low literacy are less likely to use health services [16], especially preventive services, leading to worse health outcomes and subsequently increased need for OOP expenditures [40]. The need for OOP health expenditure that arises out of poor health determine the link between the economic inequality and inequities in the delivery of health care to people in LMICs. Gender inequality and unbalanced access to education additionally contribute and sustain healthcare inequities. Affordability remains a stronger determinant for OOP health expenditure than the valid need for essential health services.

### Catastrophic health expenditure

Tanzanian households had a higher prevalence (18%) of catastrophic health expenditure at the 40% threshold, compared to the observed prevalence in Burkina Faso (10.8%) [7] and a lower prevalence than in Nigeria (27%) [41]. Our results show that a low socioeconomic status of the household increased the probability for catastrophic expenses. The association between domestic violence against women and high health expenditures are well documented [42]. Women’s welfare is vital to the household and injustice against them will affect their income contribution, health and well-being. Our finding chronic disease morbidity as an important determinant of catastrophic health expenditure has also been reported elsewhere [7]. The increased prevalence of chronic diseases in LMICs demands cost-effective control programs similar to infectious disease control programs to reduce the risk of catastrophic household health expenditure.

### Recommendations

Our study highlighted the various determinants, potentially responsible for high OOP health expenditures among the adult and older population in Tanzania. To ensure equitable health care delivery for all, OOP health expenditure should be minimized. Hence, to reduce the financial barrier and improve the accessibility to health services the following are suggested,

1. Policies to reduce OOP expenditure should extend beyond curative medical attitudes, to include preventive social welfare aspects [43].

2. Cost efficient measures focused on the vulnerable sections of the community are essential for equitable health services. Regulation of user-fees [44], cross-subsidization strategies and provision of quality medical service [45] through low operating costs for women, the physically disabled and for the elderly may ease the burden of high OOP health expenditure.

3. Community financing through prepayment schemes has demonstrated some potential for provision of financial protection to all sectors of the population[46]. However, it has been difficult to operate these schemes due to low enrolment rates and drop-outs [47]. The evaluations of strategies for scaling up are required to increase enrolment and contribute to achievement of universal coverage.

4. Traditional healers are easily available in the rural communities. Suitable training of them could influence the health care system to prevent illness, promote health and refer the sick for specialized care [48,49], which in turn would reduce the disease and financial burden. Although the adverse events are widely debated, studies argue the need for the integration of traditional healers into the health system [50].

5. Women’s health is an integral epitome of a nation’s wealth. Interventions such as provision of secondary education [51], improving the financial status of the labor division can eliminate economic barriers and enable them to control household funds. The overt financial burden faced by women due to reproductive care, childcare, along with poor incomes from manual labor can be avoided. The provision of gender sensitive health systems and entitlement to waivers can help the older women who abstain from seeking health services in spite of their significant health needs.

## Conclusion

Our study shows that the adult and older participants differ in health care demands and health service expenditures. Different factors such as economic inequality, disparities in gender and prevalence of disabilities influence the OOP health expenditures in Tanzania. Public health policies that focus on social welfare programs to make the social security systems accessible for low socioeconomic groups are essential for the establishment of equitable health services. Health care policies should consider issues such as accessibility and affordability for health services among the productive adult and vulnerable older population.

## Competing interest

All authors declare that they have no competing interests.

## Authors’ contributions

EMB, UE, AR were involved in the study design. EMB developed the research question, performed the statistical analyses and drafted the manuscript. AR contributed to the data analysis and manuscript revisions. UE supervised the study design, interpretation of results and manuscript revisions. All authors read and approved the final manuscript.

## Pre-publication history

The pre-publication history for this paper can be accessed here:

http://www.biomedcentral.com/1472-698X/14/5/prepub
