# Correction: Correlates of out-of-pocket and catastrophic health expenditures in Tanzania: results from a national household survey

**DOI:** 10.1186/1472-698X-14-18

**Published:** 2014-05-30

**Authors:** Ethel Mary Brinda, Antonio Rodríguez Andrés, Ulrika Enemark

**Affiliations:** 1 Department of Public Health, Section for Health Services Research and Health Promotion, Aarhus University, Aarhus C 8000, Denmark

## Correction

After publication of this work [1], we noted that the name and surname of one of the authors have been swapped. Now the order of names and surnames of all authors has been corrected as follows: “Ethel Mary Brinda, Antonio Rodríguez Andrés and Ulrika Enemark” (Name, surname).

## Pre-publication history

The pre-publication history for this paper can be accessed here:

http://www.biomedcentral.com/1472-698X/14/18/prepub
